# Efficacy and Safety of Qingpeng Ointment for Subacute and Chronic Eczema: A Systematic Review and Meta-Analysis

**DOI:** 10.1155/2021/5594953

**Published:** 2021-04-15

**Authors:** Yan Li, Ming Li, Boyang Zhou, Zhou Liu, Linfeng Li

**Affiliations:** Department of Dermatology, Beijing Friendship Hospital, Capital Medical University, Beijing 100050, China

## Abstract

**Objective:**

To evaluate the efficacy and safety of Qingpeng ointment for the treatment of subacute and chronic eczema.

**Method:**

Randomized controlled trials (RCTs) on Qingpeng ointment for subacute and chronic eczema were searched on PubMed, the Cochrane Library, Embase, Web of Science, China National Knowledge Infrastructure, Wanfang Database, Chinese Biomedical Literature Database, and Chinese Science and Technology Periodical Journal from their inception to 30 November 2020. Quality assessment and data analysis were performed by Review Manager 5.3.

**Results:**

A total of 26 RCTs were included. Qingpeng ointment could significantly improve the total efficacy rate (TER) (RR = 2.60, 95% CI: 2.11 to 3.21, *P* < 0.00001), reduce the total symptom score (TSS) (SMD = −2.35, 95% CI: -3.74 to -0.97, *P* = 0.0009), and decrease visual analogue scale (VAS) for pruritus (MD = −3.86, 95% CI: -4.41 to -3.31, *P* < 0.00001) compared with the placebo. The TER of Qingpeng ointment was similar to that of topical corticosteroid (TCS) (RR = 0.96, 95% CI: 0.88 to 1.03, *P* = 0.25), and the TSSs between Qingpeng ointment and medium or low potency TCS were not significantly different (SMD = −0.05, 95% CI: -0.22 to 0.12, *P* = 0.54). However, Qingpeng ointment was not superior to TCS in reducing VAS score (SMD = 0.48, 95% CI: 0.00 to 0.96, *P* = 0.05). In addition, Qingpeng ointment combined with TCS performed better than TCS alone in all three outcomes. For safety, nothing but skin irritative reactions occurred in the Qingpeng ointment group, and its incidence of skin irritative reactions was similar to those of the placebo (RR = 1.47, 95% CI: 0.61 to 3.55, *P* = 0.40) and TCS (RR = 1.82, 95% CI: 0.79 to 4.22, *P* = 0.16). The combined therapy did not increase the risk of skin irritative reactions (RR = 0.69, 95% CI: 0.27 to 1.78, *P* = 0.44).

**Conclusion:**

Qingpeng ointment is an effective and safe treatment for subacute and chronic eczema. It is also an add-on treatment to TCS for eczema. However, due to the suboptimal quality of the included studies, more large-sample and high-quality RCTs are needed to improve the evidence quality.

## 1. Introduction

Eczema is a common chronic and recurrent skin disease, which presents with a wide spectrum of skin lesions, such as erythema, papule, vesicle, scale, dry skin, and lichenification. Itch is the most important symptom [1]. In China, the prevalence of eczema in children aged 0-7 years was 18.71% [2], and the prevalence of eczema in outpatients reached 7.8% [3]. Because of skin lesions and pruritus, eczema is associated with sleep disturbances, anxiety, and depression, all of which can lead to significant psychosocial distress and economic burden [4, 5].

In many guidelines for the treatment of mild to moderate eczema, topical corticosteroids (TCSs), the standard first-line treatment, are recommended to prevent skin lesions and alleviate itch [6, 7]. However, they are associated with certain adverse effects, such as skin thinning and hyperpigmentation. The fear of side-effects of TCSs may decline adherence of patients [8], and patients with refractory chronic eczema could not be satisfied with the standard treatments. Therefore, in order to avoid adverse effects and to attain better clinical effects, many eczema patients in China have chosen to use complementary and alternative medicine.

Qingpeng ointment, a topical Tibetan medicine, has been used to treat subacute and chronic eczema in China for decades [9], and its efficacy for eczema has been proved in some studies [10, 11]. It is a mixture of Tibet herbs, including *Oxytropis falcata Bunge*, *Rheum lhasaense*, *Aconitum pendulum Busch*, *Chebulae Fructus* (without core), *Fructus Terminaliae Billericae*, *Phyllanthus emblica Linn*, Benzoin*, Tinospora sinensis (Lour.) Merr.*, and artificial musk. The herbs comprise multiple therapeutic components. For example, the main chemical ingredients of *Oxytropis falcata Bunge*, including flavonoids, alkaloids, steroids, and terpenes, show anti-inflammatory, analgesic, and antibacterial effects [12, 13]. Tannin and phenolic acids, the main chemical components of *Chebulae Fructus*, *Fructus Terminaliae Billericae*, and *Phyllanthus emblica Linn*, could reduce the release of inflammatory factors and inhibit bacteria [14].

To the best of our knowledge, there is no meta-analysis to integrate randomized controlled trials (RCTs) on Qingpeng ointment for treating eczema. Therefore, this study is aimed at systematically evaluating the efficacy and safety of Qingpeng ointment for the treatment of subacute and chronic eczema.

## 2. Materials and Methods

This study was performed according to the Preferred Reporting Items for Systematic Review and Meta-Analyses (PRISMA) guidelines [15].

### 2.1. Search Strategy

Literature search was performed in the following four English literature databases and four Chinese literature databases from their inception to 30 November 2020: PubMed, Cochrane Library, Embase, Web of Science, China National Knowledge Infrastructure (CNKI), Wanfang Database (Wanfang), Chinese Biomedical Literature Database (Sinomed), and Chinese Science and Technology Periodical Journal (VIP). The search strategies used the following words and MeSH terms: ((“eczema” [MeSH] OR “dermatitis” [MeSH]) OR (“eczema” [Title/Abstract] OR “dermatitis” [Title/Abstract])) AND (“Qingpeng” [Title/Abstract] OR “Qing Peng” [Title/Abstract]).

### 2.2. Inclusion and Exclusion Criteria

#### 2.2.1. Types of Study Design

RCTs published in Chinese or English were included.

#### 2.2.2. Types of Participants

The patients were diagnosed as subacute eczema or chronic eczema, regardless of age, gender, disease course, and disease severity. The inclusion and exclusion criteria on the types of eczema are listed in Table 1.

#### 2.2.3. Types of Interventions

The participants in the test group were treated with Qingpeng ointment alone or Qingpeng ointment combined with TCS.

#### 2.2.4. Types of Comparisons

The participants in the control group were treated with the placebo or TCS.

#### 2.2.5. Types of Outcomes

The primary outcomes were the total efficacy rate (TER) and total symptom score (TSS). TSS was scored based on the severities of lesion morphology, lesion area, and pruritus symptom. In addition, complete cure was defined as the decreased proportion of TSS ≥ 90%; significant improvement was defined as 60% ≤ the decreased proportion of TSS < 89%; moderate improvement was defined as 20% ≤ the decreased proportion of TSS < 59%; no improvement was defined as the decreased proportion of TSS < 20%. TER = (the number of patients with complete cure + the number of patient with significant improvement)/total number of patients × 100%.

The second outcomes included visual analogue scale (VAS) for pruritus and adverse events. Among skin adverse events, erythema, itch, pain, tingling, burning, etc. belonged to skin irritative reactions, and skin non-irritative reactions included hyperpigmentation, hypertrichosis, skin infection, atrophy, and telangiectasia.

Reviews, animal experiments, case reports, duplicates, unavailable full texts, and studies with inappropriate interventions were excluded.

#### 2.2.6. Literature Selection and Data Extraction

Based on the inclusion and exclusion criteria, the eligible studies were selected by reading the titles, abstracts, and full-texts of studies. The following data were extracted from each included study: the first author's name, year of publication, sample size, age, the interventions of each group, the treatment course, and outcome measures.

### 2.3. Quality Assessment

The methodological quality of the included studies was evaluated based on the Cochrane Collaboration Risk of Bias Tool [16]. The risk of bias included the following seven items: random sequence generation, allocation concealment, blinding of participants and personnel, blinding of outcome assessment, incomplete outcome data, selective reporting, and other bias. In this study, the baselines of disease severity between the two groups were considered as the source of other bias. Each item of each RCT was classified as low, unclear, or high risk of bias. The quality of evidence was assessed by using the Grading of Recommendations Assessment, Development and Evaluation (GRADE) system [17]. The level of evidence was classified into four grades: very low, low, moderate, and high, and the level was assessed based on the following five factors: study limitations, inconsistency of results, indirectness of evidence, imprecision, and reporting bias.

### 2.4. Statistical Analyses

All statistical analyses were performed by the Review Manager 5.3. Dichotomous data were expressed as relative risk (RR) with 95% confidence interval (CI), whereas continuous data were expressed as mean difference (MD) or standardized mean difference (SMD) with 95% CI. The heterogeneity across studies was tested by *I*^2^ statistic. A fixed-effects model (*I*^2^ < 50%) or a random effects model (*I*^2^ ≥ 50%) was used depending on the value of *I*^2^. The differences between both groups were considered to be statistically significant when *P* < 0.05. The publication bias was evaluated by using the funnel plot if the number of included studies was 10 or more.

## 3. Results

### 3.1. Study Selection

**Figure 1:**
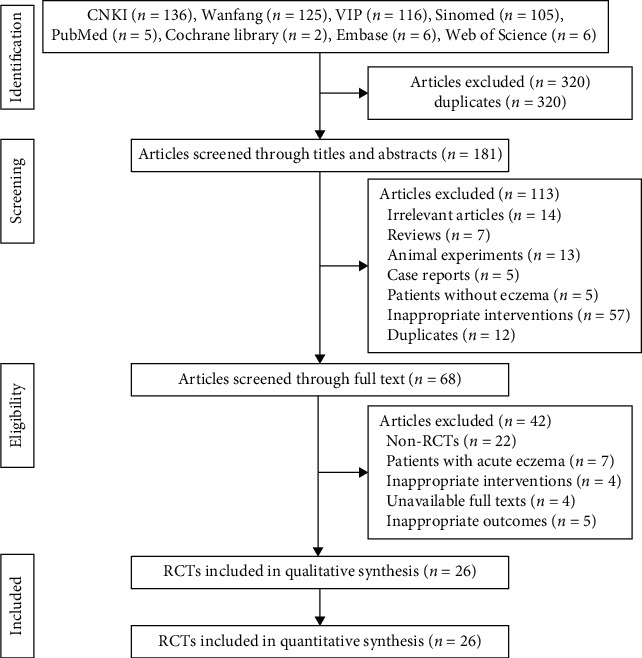
Flowchart of the study selection process.

A total of 501 studies were identified from the four English literature databases and four Chinese literature databases. After removing the duplicates, 181 studies remained for further selection. 113 studies were excluded based on the titles and abstracts, and 42 studies were removed according to the full texts. Ultimately, 26 eligible studies were included in the meta-analysis [18–43]. A flowchart of the study selection process is summarized in Figure 1.

### 3.2. Characteristics of Included Studies

All studies were published between 2010 and 2019 and were conducted in China. Only one study was published in English [18], while the rest were in Chinese [19–43]. Apart from one multicenter trial [19], the rest of the 25 studies were single-center trials. The sample size of the studies ranged from 44 to 244. Moreover, the participants of 5 studies were children [20–22, 26, 37]. There were 3 three-arm trials [23, 32, 33] and 23 two-arm trials. Among 26 studies, 7 studies compared Qingpeng ointment with the placebo [18–24], 13 studies compared Qingpeng ointment with TCS [23, 25–36], and 9 studies compared Qingpeng ointment plus TCS with TCS [32, 33, 37–43]. The characteristics of the included studies are listed in Table 2.

### 3.3. Risk of Bias of Included Studies

**Figure 2:**
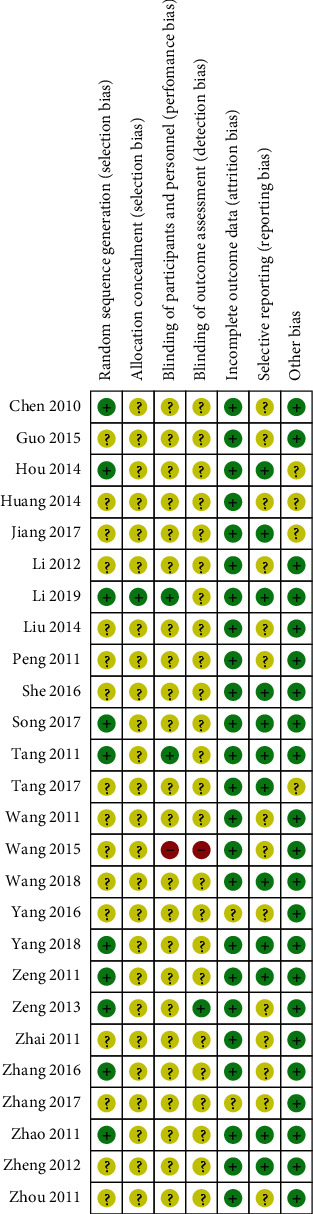
The risk of bias of the included studies.

All studies declared randomization, however, only 10 studies provided the details of random sequence generation, including one study with throwing dice [36], one study with computer-generated random number [18], and 8 studies with a list of random numbers [19, 23, 24, 26, 27, 31, 34, 37]. Moreover, all but one study did not describe allocation concealment [18]. Among 26 trials, two trials and one trial mentioned blinding of participants and personnel and blinding of outcome assessment, respectively [18, 19, 23], whereas blinding bias was at high risk in one open-labeled trial [39]. In addition, only two studies did not describe the reasons for incomplete data [21, 28]. Reporting bias was at low risk in 12 studies [18–20, 22, 25, 26, 31, 33, 34, 36–38], and it was unclear whether the rest of the studies had selective reporting. For other bias, the baseline data of eczema severities between two groups were not significantly different in 22 studies, and the remaining 4 studies did not provide the baseline data [25, 30, 31, 38]. The risk of bias summary is shown in Figure 2.

### 3.4. Primary Outcomes

#### 3.4.1. Total Efficacy Rate

**Figure 3:**
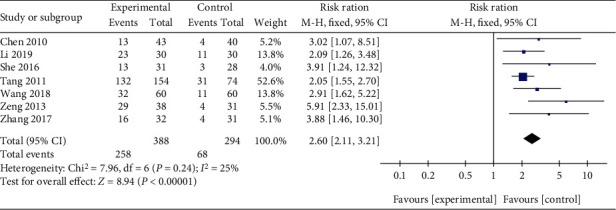
Forest plot of the TER between Qingpeng ointment and the placebo.

Seven trials assessed the TER between Qingpeng ointment and the placebo [18–24]. Because of little heterogeneity (*I*^2^ = 25%, *P* = 0.24), a fix-effect model was employed. The pooled results indicated that Qingpeng ointment significantly increased the TER compared with the placebo (RR = 2.60, 95% CI: 2.11 to 3.21, *P* < 0.00001), as shown in Figure 3.

**Figure 4:**
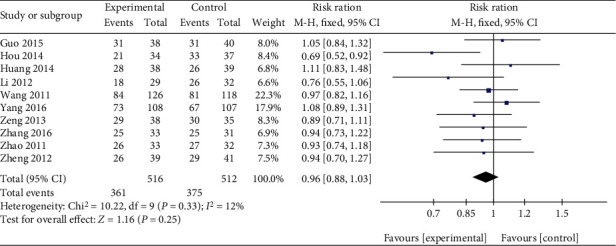
Forest plot of the TER between Qingpeng ointment and TCS.

Ten trials reported the TER between Qingpeng ointment and TCS [23, 27–35]. No significant heterogeneity was detected (*I*^2^ = 12%, *P* = 0.33), and a fix-effect model was used. The pooled results showed there was no significant difference in TER between Qingpeng ointment and TCS (RR = 0.96, 95% CI: 0.88 to 1.03, *P* = 0.25), as shown in Figure 4.

**Figure 5:**
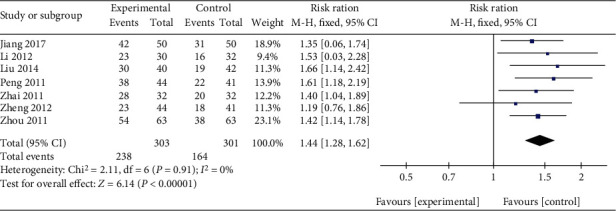
Forest plot of the TER between Qingpeng ointment plus TCS and TCS.

Seven trials compared Qingpeng ointment plus TCS with TCS alone in terms of TER [32, 33, 38, 40–43]. Due to little heterogeneity (*I*^2^ = 0%, *P* = 0.91), a fix-effect model was applied. The pooled results indicated that patients receiving Qingpeng ointment combined with TCS had a significantly higher TER than those receiving TCS alone (RR = 1.44, 95% CI: 1.28 to 1.62, *P* < 0.00001), as shown in Figure 5.

#### 3.4.2. Total Symptom Score

**Figure 6:**
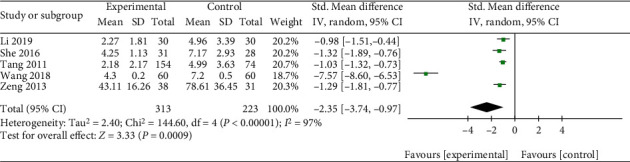
Forest plot of the TSS between Qingpeng ointment and the placebo.

The TSS of Qingpeng ointment and the placebo was reported in five studies [18–20, 22, 23]. Because of different evaluation methods, SMD with 95% CI was applied. A random-effect model was used due to the apparent heterogeneity among the studies (*I*^2^ = 97%, *P* < 0.00001). The pooled results suggested that the TSS of the Qingpeng ointment group decreased more significantly than that of the placebo group (SMD = −2.35, 95% CI: -3.74 to -0.97, *P* = 0.0009), as shown in Figure 6.

**Figure 7:**
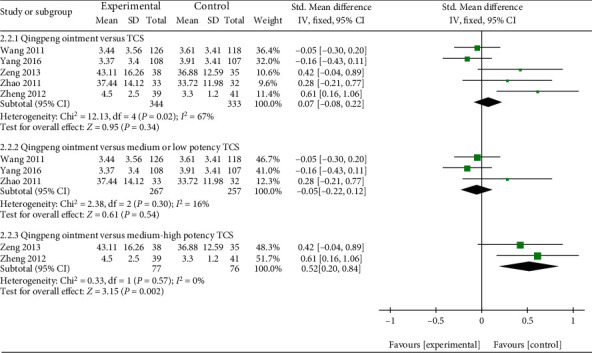
Forest plot of the TSS between Qingpeng ointment and TCS.

Five studies provided available data on the TSS of Qingpeng ointment and TCS [23, 28, 33–35]. Due to the different methods of assessing TSS, SMD with 95% CI was employed. High heterogeneity was observed among the studies (*I*^2^ = 67%, *P* = 0.02), and a subgroup analysis was conducted based on the potency ranking of TCS. The result revealed that in term of reducing TSS, there was no significant difference between Qingpeng ointment and medium or low potency TCS (SMD = −0.05, 95% CI: -0.22 to 0.12, *P* = 0.54), however, Qingpeng ointment was inferior to medium-high potency TCS (SMD = 0.52, 95% CI: 0.20 to 0.84, *P* = 0.002), as shown in Figure 7.

**Figure 8:**
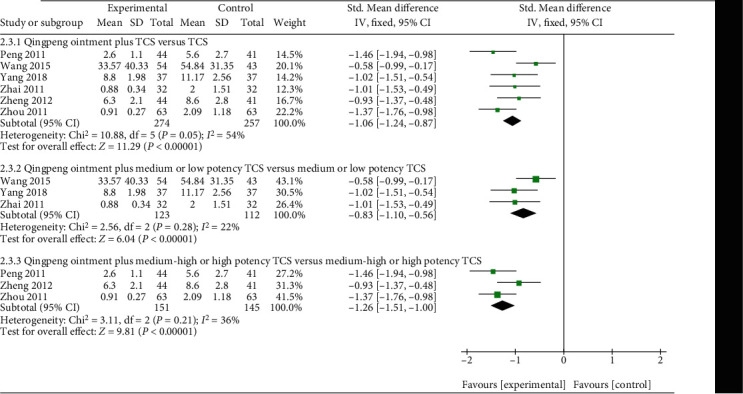
Forest plot of the TSS between Qingpeng ointment plus TCS and TCS.

Six studies mentioned the TSS of Qingpeng ointment plus TCS and TCS alone [33, 37, 39, 41–43]. Because of different standards of TSS, SMD with 95% CI was used. High heterogeneity among the studies (*I*^2^ = 54%, *P* = 0.05) was observed, and a subgroup analysis was performed based on the potency ranking of TCS. The result displayed that the combination of Qingpeng ointment and medium or low potency TCS was more effective than medium or low potency TCS alone in terms of reducing TSS (SMD = −0.83, 95%CI: -1.10 to -0.56, *P* < 0.00001). A similar result was also applicable for the Qingpeng ointment combined with medium-high or high potency TCS group (SMD = −1.26, 95%CI: -1.51 to -1.00, *P* < 0.00001), as shown in Figure 8.

### 3.5. Secondary Outcomes

#### 3.5.1. Visual Analogue Scale for Pruritus

**Figure 9:**
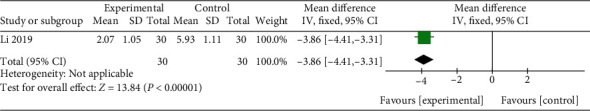
Forest plot of the VAS score between Qingpeng ointment and the placebo.

The data on the VAS score of Qingpeng ointment and the placebo were collected in one study [18]. The result showed that Qingpeng ointment was effective in alleviating pruritus (MD = −3.86, 95% CI: -4.41 to -3.31, *P* < 0.00001), as shown in Figure 9.

**Figure 10:**
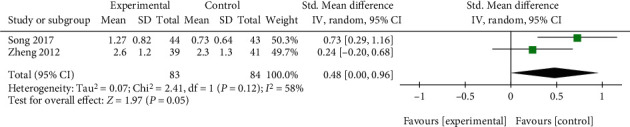
Forest plot of the VAS score between Qingpeng ointment and TCS.

Two studies assessed the VAS score of Qingpeng ointment and TCS [26, 33]. The ranges of VAS score between the two studies were different, and SMD with 95% CI was conducted. Due to high heterogeneity (*I*^2^ = 58%, *P* = 0.12), a random-effect model was used. The pooled results showed that Qingpeng ointment was not superior to TCS in relieving pruritus (SMD = 0.48, 95% CI: 0.00 to 0.96, *P* = 0.05), as shown in Figure 10.

**Figure 11:**
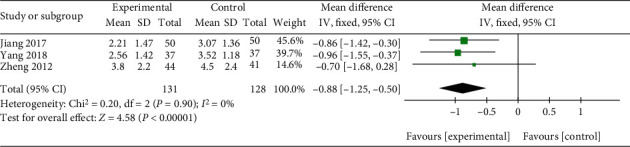
Forest plot of the VAS score between Qingpeng ointment plus TCS and TCS.

Three studies compared the VAS score of Qingpeng ointment plus TCS with that of TCS alone [33, 37, 38]. MD with 95% CI was used due to the same range of VAS score across three studies. No heterogeneity was observed (*I*^2^ = 0%, *P* = 0.90), and a fix-effect model was conducted. The pooled results showed that the combination of Qingpeng ointment and TCS could alleviate itch better than TCS alone (MD = −0.88, 95% CI: -1.25 to -0.50, *P* < 0.00001), as shown in Figure 11.

#### 3.5.2. Adverse Event

In the studies which reported adverse event, nothing but skin adverse events were observed in each group during the treatment.

**Figure 12:**
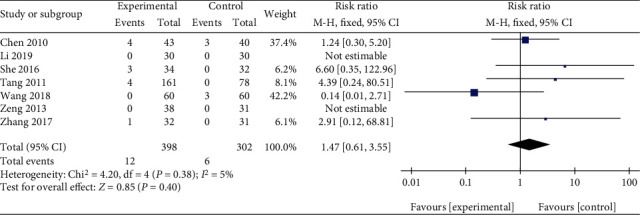
Forest plot of the skin irritative reactions between Qingpeng ointment and the placebo.

Among seven studies comparing Qingpeng ointment with the placebo [18–24], no adverse event was observed in 129 patients in two studies [18, 23]. The remaining five studies reported skin adverse events, and only skin irritative reactions were discovered [19–22, 24]. Because of little heterogeneity (*I*^2^ = 5%, *P* = 0.38), a fix-effect model was applied. The pooled results showed that Qingpeng ointment did not significantly increase the incidence of skin irritative reactions compared with the placebo (RR = 1.47, 95% CI: 0.61 to 3.55, *P* = 0.40), as shown in Figure 12.

**Figure 13:**
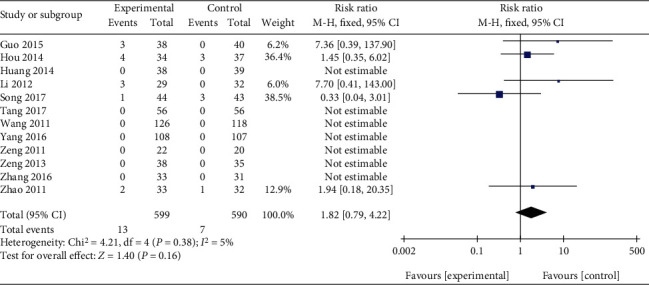
Forest plot of the skin irritative reactions between Qingpeng ointment and TCS.

Twelve studies comparing Qingpeng ointment with TCS offered available data on adverse events [23, 25–32, 34–36]. Among 1189 participants, 13 cases in the Qingpeng group and 7 cases in the TCS group experienced some skin irritative reactions during the treatment. Little heterogeneity among studies was observed (*I*^2^ = 5%, *P* = 0.38), and a fix-effect model was used. The pooled results revealed that the risk of skin irritative reactions in the Qingpeng ointment group was similar to that in the TCS group (RR = 1.82, 95% CI: 0.79 to 4.22, *P* = 0.16), as shown in Figure 13. On the other hand, no patient in the Qingpeng group experienced skin non-irritative reactions, while 19 cases of skin non-irritative reactions were observed in the TCS group [25, 29, 32], including 11 cases of hyperpigmentation, 3 cases of skin infection, 2 cases of skin atrophy, and 3 cases of telangiectasia.

**Figure 14:**
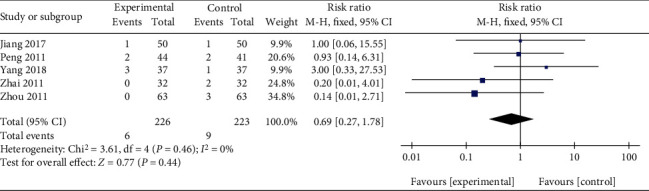
Forest plot of the skin irritative reactions between Qingpeng ointment plus TCS and TCS.

The available data on adverse events were provided in five studies comparing Qingpeng ointment plus TCS with TCS [37, 38, 41–43]. Skin irritative reactions were observed in all five studies, and one study reported a case of hyperpigmentation in the TCS group [38]. Due to little heterogeneity (*I*^2^ = 0%, *P* = 0.46), a fix-effect model was applied. The pooled results showed that the combination of Qingpeng ointment and TCS did not increase the incidence of skin irritative reactions compared with TCS alone (RR = 0.69, 95% CI: 0.27 to 1.78, *P* = 0.44), as shown in Figure 14.

### 3.6. Sensitivity Analysis and Publication Bias

**Figure 15:**
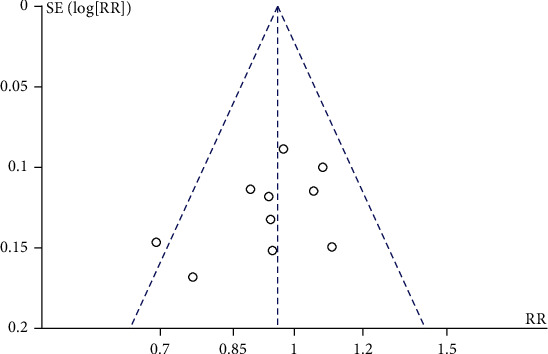
Funnel plot of publication bias for the TER between Qingpeng ointment and TCS.

The sensitivity analysis was performed in each meta-analysis, and it found that any individual study could not significantly affect the pooled results, indicating a high stability of the analysis. Publication bias on the TER of Qingpeng ointment and TCS was assessed. The nearly symmetrical funnel plot suggested no obvious publication bias, as shown in Figure 15.

### 3.7. GRADE Evaluation

As the included studies had the suboptimal methodological quality, such as the absence of blinding and allocation concealment, the level of evidence was moderate for TER and adverse events. On the other hand, due to the suboptimal methodological quality, sample size less than 400, and the significant heterogeneity across studies, the level of evidence was very low to moderate for TSS and VAS for pruritus. The GRADE summary of the included studies is shown in Table 3.

## 4. Discussion

Qingpeng ointment has been widely used for the treatment of subacute and chronic eczema for decades, and a lot of clinical trials have demonstrated its efficacy and safety. However, to date, there is no evidence-based medical proof to support Qingpeng ointment for treating eczema. Therefore, this is the first meta-analysis collecting 26 RCTs to investigate the efficacy and safety of Qingpeng ointment for subacute and chronic eczema.

The pathogenesis of eczema is complex, including immune dysregulation and epidermal barrier dysfunction [44]. Some animal experiments investigated the anti-inflammatory effect of Qingpeng ointment in atopic dermatitis-like murine model, and they revealed that Qingpeng ointment could not only inhibit the infiltration CD4^+^ T cells and mast cells in the dermis of the lesion but also suppress the production of IL-4 and the mRNA expression of IL-17A in tissue, while the levels of IFN-*γ* and TNF-*α* in tissue were increased [45, 46]. In addition, as some clinical trials showed, Qingpeng ointment could significantly increase the water content of the stratum corneum and decrease transepidermal water loss [47–49]. Therefore, Qingpeng ointment is able to modulate the immune dysfunctions and to restore the skin barrier function. In this meta-analysis, we found that Qingpeng ointment could significantly improve the TER and decrease the TSS score compared with the placebo, indicating that Qingpeng ointment is an effective topical treatment for subacute and chronic eczema. On the other hand, in comparison with positive control medicine, the TER of Qingpeng ointment and TCS was similar, and there was no significant difference in the TSS between Qingpeng ointment and medium or low potency TCS, while medium-high potency TCS performed better in reducing the TSS than Qingpeng ointment. These different results between TER and TSS may be attributed to the fact that all included participants were patients with mild to moderate eczema, and TSS may be more suitable than TER for distinguishing small changes in the efficacy of Qingpeng ointment. The above data also demonstrated the efficacy of Qingpeng ointment and showed that it might be an alternative to low and medium potency TCS for treating eczema. However, the finding should be interpreted with caution due to a few studies and limited sample size.

In addition, itch is a remarkable manifestation of eczema, and the treatment of itch is still a great challenge for clinicians and patients. Some animal experiments investigated the antipruritic effect of Qingpeng ointment by using the histamine-induced itch mice model, and the results showed that Qingpeng ointment could significantly decrease thymic stromal lymphopoietin (TSLP) mRNA and IL-4 mRNA levels in the skin and increase the pruritus threshold of histamine phosphate [50, 51]. Another trial used the squaric acid dibutylester-induced allergic contact dermatitis mice model and showed that Qingpeng ointment can attenuate scratching behavior by reversing the upregulation of mRNA levels of itch-related genes and inhibiting the phosphorylation of mitogen-activated protein kinases (MAPKs) in the skin [9]. In this meta-analysis, Qingpeng ointment could apparently reduce more VAS score than sham ointment, suggesting its ability of alleviating pruritus. However, two studies showed that the VAS score of the Qingpeng ointment group was not significantly lower than that of the TCS group. Because a few studies were included, more studies are needed to further confirm the antipruritic effect of Qingpeng ointment and to compare Qingpeng ointment with different potency TCS for relieving itch.

On the other hand, many patients with refractory eczema are not satisfied with the effect of TCS alone and are willing to be treated by TCS combined with traditional medicine. In this meta-analysis, compared with TCS alone, Qingpeng ointment combined with TCS could not only remarkably improve the TER but also significantly reduce both TSS and VAS score. In clinical practice, the combination of Qingpeng ointment and TCS could reduce excess application of TCS and avoid the side effects of TCS. Therefore, Qingpeng ointment is an add-on treatment to TCS for treating eczema, and the combination of Qingpeng ointment and TCS might be an optional treatment for patients with refractory eczema.

In clinical practice, drug safety is as important as its efficacy. Our study revealed that no serious adverse events but skin irritative reactions were reported in the Qingpeng ointment groups, including erythema, itch, and burning. The symptoms could disappear spontaneously after drug withdrawal. In this meta-analysis, the incidence of skin irritative reactions of Qingpeng ointment was similar to those of the placebo and TCS. Meanwhile, the combined therapy did not increase the risk of skin irritative reactions. In addition, the common side effects of TCS, such as hyperpigmentation and skin thinning, were not observed in the patients treated with Qingpeng ointment. Therefore, Qingpeng ointment is a safe topical medicine. However, some laboratory examinations were not conducted during the treatment in all included studies, such as blood routine test, urine routine test, hepatic function, and renal function. Although no serious adverse event during four weeks of treatment is observed, we need be cautious of the safety of long-term use of Qingpeng ointment, and regular laboratory examinations are needed in future studies to confirm the safety of Qingpeng ointment for other organs, such as liver and kidney.

There are some limitations in this meta-analysis. Firstly, the methodological qualities of the included studies were low. Most studies failed to provide the details of allocation concealment, blinding methods, and sequence generation methods, which could increase the risk of bias and degrade the level of evidence. Secondly, all trials were conducted in China. There is a lack of clinical evidence for other races. Finally, eczema is a chronic recurrent skin disease, and the therapeutic goal is to maintain the efficacy of treatment and to reduce the relapse of lesions. However, few trials have a long-term follow-up to observe Qingpeng ointment in decreasing recurrence rate. Therefore, well-designed and large-sample RCTs are warranted to further explore the long-term efficacy and safety of Qingpeng ointment for the treatment of eczema.

## 5. Conclusions

Our study reveals that Qingpeng ointment is an effective and safe treatment for subacute and chronic eczema. It might be an alternative to medium and low potency TCS for reducing eczema severity, and it is also an add-on treatment to TCS for treating eczema. However, these findings should be interpreted carefully because of the poor methodological quality and limited sample size. More high-quality, large-sample RCTs are needed to provide high-quality evidence of Qingpeng ointment for eczema in the future.

## Figures and Tables

**Table 1:** The inclusion and exclusion on the types of eczema^∗^.

The included types of eczema	The excluded types of eczema
Atopic eczema	Allergic contact dermatitis
Seborrheic dermatitis	Photo-allergic contact dermatitis
Nummular dermatitis	Irritant contact dermatitis
Lichen simplex chronicus	Eczema of eyelids
Asteatotic eczema	Eczema of external ear
Eczema of hands and feet	Eczematous nail dystrophy
Eczema of lower legs	
Eczema of anogenital region	
Unspecified eczema	

^∗^The types of eczema are classified based on the International Classification of Disease-11 (ICD-11).

**Table 2:** Characteristics of all included studies.

First author	Sample size (T/C)	Age (years)		Interventions		Duration treatment (weeks)	Outcomes
		T	C	T	C		
Li 2019	60 (30/30)	26-72	20-74	QP	Sham ointment	2	①②③④
Tang 2011	228 (154/74)	18-70	18-70	QP	Sham ointment	3	①②④
Wang 2018	120 (60/60)	2-11	2-12	QP	Vaseline ointment	2	①②④
Zhang 2017	67 (34/33)	2-12	3-12	QP	Vaseline ointment	2	①④
She 2016	65 (33/32)	2-12	2-12	QP	Vaseline ointment	2	①②④
Zeng^∗^ 2013	69 (38/31)	20-63	20-63	QP	Vaseline ointment	4	①②④
Chen 2010	83 (43/40)	29-68	31-69	QP	Vaseline ointment	4	①④
Tang 2017	112 (56/56)	22-64	21-63	QP	Triamcinolone acetonide cream	2	④
Song 2017	87 (44/43)	4-12	4-12	QP	Hydrocortisone butyrate ointment	4	③④
Zhang 2016	64 (33/31)	18-60	18-60	QP	Hydrocortisone butyrate ointment	4	①④
Yang 2016	215 (108/107)	18-64	17-58	QP	Hydrocortisone butyrate ointment	2	①②④
Guo 2015	78 (38/40)	15-70	16-68	QP	Halometasone cream	4	①④
Huang 2014	77 (38/39)	18-65	18-65	QP	Hydrocortisone butyrate ointment	3	①④
Hou 2014	71 (34/37)	18-72	18-72	QP	Hydrocortisone butyrate ointment	3	①④
Zeng^∗^ 2013	73 (38/35)	20-63	20-63	QP	Mometasone furoate cream	4	①②④
Li^∗^ 2012	61 (29/32)	18-69	18-75	QP	Fluticasone propionate cream	4	①④
Zheng^∗^ 2012	80 (39/41)	25-70	21-65	QP	Mometasone furoate cream	3	①②③
Zhao 2011	65 (33/32)	15-86	15-86	QP	Hydrocortisone cream	4	①②④
Wang 2011	244 (126/118)	18-65	16-60	QP	Hydrocortisone butyrate ointment	2	①②④
Zeng 2011	44 (22/20)	18-75	18-75	QP	Hydrocortisone butyrate ointment	4	④
Yang 2018	74 (37/37)	4-10	4-9	QP + desonide ointment	Desonide ointment	4	②③④
Jiang 2017	100 (50/50)	19-78	20-81	QP + mometasone furoate cream	Mometasone furoate cream	2	①③④
Wang 2015	97 (54/43)	35.69	34.54	QP + hydrocortisone butyrate ointment	Hydrocortisone butyrate ointment	2	②
Liu 2014	82 (40/42)	21-70	19-72	QP + halometasone cream	Halometasone cream	2	①
Li^∗^ 2012	62 (30/32)	21-72	18-75	QP + fluticasone propionate cream	Fluticasone propionate cream	2	①
Zheng^∗^ 2012	85 (44/41)	18-72	21-65	QP + mometasone furoate cream	Mometasone furoate cream	1	①②③
Zhou 2011	126 (63/63)	15-70	15-70	QP + halometasone cream	Halometasone cream	2	①②④
Zhai 2011	64 (32/32)	16-64	16-64	QP + fluticasone propionate cream	Fluticasone propionate cream	2	①②④
Peng 2011	85 (44/41)	19-63	18-64	QP + triamcinolone acetonide cream	Triamcinolone acetonide cream	4	①②④

T: test group; C: control group; QP: Qingpeng ointment; ①: total efficacy rate; ②: total symptom score; ③: visual analogue scale for pruritus; ④: adverse events. ^∗^A three-arm study.

**Table 3:** GRADE summary of included studies.

Outcomes	Intervention	Effect		Number of participants (studies)	Quality of the evidence (GRADE)
		Relative effect (95% CI)	Absolute effect (95%)		
TER	QP versus placebo	RR:2.60 (2.11 to 3.21)	370 more per 1000 (from 257 more to 511 more)	682 (7 studies)	⊕⊕⊕⊖ Moderate^a^
	QP versus TCS	RR:0.96 (0.88 to 1.03)	29 fewer per 1000 (from 88 fewer to 22 more)	1028 (10 studies)	⊕⊕⊕⊖ Moderate^a^
	QP + TCS versus TCS	RR:1.44 (1.28 to 1.62)	240 more per 1000 (from 153 more to 338 more)	604 (7 studies)	⊕⊕⊕⊖ Moderate^a^
TSS	QP versus placebo	—	SMD:2.35 lower (3.74 lower to 0.97 lower)	536 (5 studies)	⊕⊕⊖⊖ Low^a,b^
	QP versus TCS^1^	—	SMD: 0.05 lower (0.22 lower to 0.12 higher)	524 (3 studies)	⊕⊕⊕⊖ Moderate^a^
	QP versus TCS^2^	—	SMD: 0.52 higher (0.20 higher to 0.84 higher)	153 (2 studies)	⊕⊕⊖⊖ Low^a,c^
	QP + TCS versus TCS^3^	—	SMD:0.83 lower (1.10 lower to 0.56 lower)	235 (3 studies)	⊕⊕⊖⊖ Low^a,c^
	QP + TCS versus TCS^4^	—	SMD: 1.26 lower (1.51 lower to 1.00 lower)	296 (3 studies)	⊕⊕⊖⊖ Low^a,c^
VAS for pruritus	QP versus placebo	—	MD: 3.86 lower (4.41 lower to 3.31 lower)	60 (1 study)	⊕⊕⊖⊖ Low^a,c^
	QP versus TCS	—	SMD: 0.48 higher (0.00 to 0.96 higher)	167 (2 studies)	⊕⊖⊖⊖ Very Low^a,b,c^
	QP + TCS versus TCS	—	MD: 0.88 lower (1.25 lower to 0.50 lower)	259 (3 studies)	⊕⊕⊖⊖ Low^a,c^
Skin irritative reaction	QP versus placebo	RR:1.47 (0.61 to 3.55)	9 more per 1000 (from 8 fewer to 51 more)	700 (7 studies)	⊕⊕⊕⊖ Moderate^a^
	QP versus TCS	RR:1.82 (0.79 to 4.22)	10 more per 1000 (from 2 fewer to 38 more)	1189 (12 studies)	⊕⊕⊕⊖ Moderate^a^
	QP + TCS versus TCS	RR:0.69 (0.27 to 1.78)	13 fewer per 1000 (from 29 fewer to 31 more)	449 (5 studies)	⊕⊕⊕⊖ Moderate^a^

TER: total efficacy rate; TSS: total symptom score; VAS: visual analogue scale; QP: Qingpeng ointment; TCS: topical corticosteroid; ^1^Qingpeng ointment versus medium or low potency TCS; ^2^Qingpeng ointment versus medium-high potency TCS; ^3^Qingpeng ointment combined with medium or low potency TCS versus medium or low potency TCS; ^4^Qingpeng ointment combined with medium-high or high potency TCS versus medium-high or high potency TCS; ^a^suboptimal methodological quality; ^b^the significant heterogeneity among the studies; ^c^the total number of events was less than 400.

## Data Availability

The datasets generated and analyzed during the present study are available from the corresponding author on reasonable request.
